# Supplementary material for: Examining the Transcriptomic and Biochemical Signatures of Bacillus subtilis Strains: Impacts on Plant Growth and Abiotic Stress Tolerance
Source: Int J Mol Sci. 2023 Sep 6;24(18):13720. doi: 10.3390/ijms241813720 (PMC10531026; doi:10.3390/ijms241813720)
Supplement: Supplementary file 1 [file ijms-24-13720-s001.zip › ijms-2579019-supplementary.pdf]

Supplemental Table 1. Primer sequences used for PCR and qPCR analysis

For PCR analysis

Primer	Sequence	Locus
fD1	5’-AGAGTTTGATCCTGGCTCAG-3’	AB257199
rP1	5’-ACGGTTACCTTGTTACGACTT-3’	AB257199
DhbF	F: 5’-GAGGATTGGTACAACCGCCA-3’	AEW31033
	R: 5’-GCTAACCCCGCTCCTGATAC-3’	AEW31033
DhbB	F: 5’-TCAGCCGTATCAAATGCCGA-3’	AAC44633
	R: 5’-AGGACCGCTGTTTAATCCCG-3’	AAC44633
DhbE	F: 5’-GCCTCTTGCGATGGTATGGA-3’	AAC44632
	R: 5’-CTCTGCCATTCCGAACACCT-3’	AAC44632
sfp	F: 5’-ATGAAGATTTACGGAATTTA-3’	EU882341
	R: 5’-TTATAAAAGCTCTTCGTACG-3’	EU882341

For qPCR analysis

Primer	Sequence	Locus
ACT2	F: 5’-CGGTAACATTGTGCTCAGTG-3’	AT3G18780
	R: 5’-GTGAACGATTCCTGGACCTG-3’	AT3G18780
ICS1	F: 5’GGCTGCTCTGCATCCAACTC-3’	AT1G74710
	R:5’- CGCCTGTAGAGATGTTGTTG-3’	AT1G74710
EDS1	F: 5’-GAATTCGGGATCCGAGTGCG-3’	AT3G48090
	R: 5’-CCATCATATAGTCTCGCAGAG-3’	AT3G48090
CBP60G	F: 5’-GTGAACTCGATCAAGTACAC-3’	AT5G26920
	R: 5’-CTGGCAGTTGTGTGTCTCCG-3’	AT5G26920
LOX1	F: 5’-CGGACAGTATCCAGTTGCTG-3’	AT1G55020
	R: 5’-GTTCTTGAGAGTGTCGTCGT-3’	AT1G55020
OPR3	F: 5’-GGAACTAGGTATGCAAGCTG-3’	AT2G06050
	R: 5’-CAACAACTGGATCTTGAGTG-3’	AT2G06050
MYC2	F: 5’-GCGAGTGCTAGTGGAGGAGA-3’	AT1G32640
	R: 5’-GCTCTGAGCTGTTCTTGCGT-3’	AT1G32640
NCED3	F: 5’-CGGTGGTTTACGACAAGAAC-3’	AT3G14440
	R: 5’-CCATCCCTGCTTCGAGGTTG-3’	AT3G14440
ABI5	F: 5’-GGATTAGGACATGGACAAGT-3’	AT2G36270
	R: 5’-GTGGACAACTCGGGTTCCTC-3’	AT2G36270
SDR4	F: 5’-GCTTCTAAGCACGCGCTTCT-3’	AT3G29250
	R: 5’-TCATGAGCTTAACGACGCTA-3’	AT3G29250
DREB2A	F: 5’-ATGGCAGTTTATGATCAGAG-3’	AT5G05410
	R: 5’-GTTCTCCAGATCCAAGTAAC-3’	AT5G05410
ZAT12	F: 5’-GAGACACAGGAACGAGAGTG-3’	AT5G59820
	R: 5’-CTGTTCTTCCAAGCTCCAAC-3’	AT5G59820
APX1	F: 5’-GACCATTTGAGAGATGTC-3’	AT1G07890
	R: 5’-AGCATCAGCAAACCCAAG-3’	AT1G07890
GPX7	F: 5’-CCGGTTCAAAGCAGAGTTCC-3’	AT4G31870
	R: 5’-TTGTGGGAGGGTATCTCTCG-3’	AT4G31870
P5CS1	F: 5’-GGACTTGGTGCAGAGGTGGG-3’	AT2G39800
	R: 5’-GCTTGGATGGGAATGTCCTG-3’	AT2G39800
CAB1	F: 5’-GGAAGATTGGCTATGTTCTC-3’	AT1G29930
	R: 5’-CGGGAACAAAGTTGGTGGCG-3’	AT1G29930
FRK1	F: 5’-CAGGCCAACCTGCTATTGCA-3’	AT2G19190
	R: 5’-GGAACCATCTCGGTGTCTAG-3’	AT2G19190
PR1	F: 5’-GGCAACTGCAGACTCATACA-3’	AT2G14610
	R: 5’-GTATGGCTTCTCGTTCACAT-3’	AT2G14610
PR2	F: 5’-ATGTCTGAATCAAGGAGCTT-3’	AT3G57260
	R: 5’-GTGGTGGTGTCAGTGGCTAT-3’	AT3G57260
THI2.1	F: 5’-CTGCCCTTCCAACCAAGCTA-3’	AT1G72260
	R: 5’-TTGTTCCGACGCTCCATTCA-3’	AT1G72260
NIT2	F: 5’-CGTTTACGACACTCCGATTG-3’	AT3G44300
	R: 5’-CTGGTCTCGAGTAATGTCCA-3’	AT3G44300
RBOHC	F: 5’-CAAGCGTATAGCTATGGATC-3’	AT5G51060
	R: 5’-GTGGAAGGAGAATCTGGTGC-3’	AT5G51060

Source of database:

Genes analyzed in PCR: https://www.ncbi.nlm.nih.gov/

Genes analyzed in qPCR: https://www.arabidopsis.org/
